# Characteristics, outcomes, and risk factors for in-hospital mortality of COVID-19 patients: A retrospective study in Thailand

**DOI:** 10.3389/fmed.2022.1061955

**Published:** 2023-01-04

**Authors:** Thummaporn Naorungroj, Tanuwong Viarasilpa, Surat Tongyoo, Aeckapholpholladet Detkaew, Thanchanok Pinpak, Rawish Wimolwattanaphan, Ranistha Ratanarat, Panuwat Promsin, Preecha Thamrongpiroj, Akekarin Phumpichet, Chairat Permpikul

**Affiliations:** Division of Critical Care, Department of Medicine Siriraj Hospital, Mahidol University, Bangkok, Thailand

**Keywords:** mortality, coronavirus, COVID-19, SARS-CoV-2, risk factor, Thailand, developing country

## Abstract

**Introduction:**

Data on the characteristics and outcomes of patients hospitalized for Coronavirus Disease 2019 (COVID-19) in Thailand are limited.

**Objective:**

To determine characteristics and outcomes and identify risk factors for hospital mortality for hospitalized patients with COVID-19.

**Methods:**

We retrospectively reviewed the medical records of patients who had COVID-19 infection and were admitted to the cohort ward or ICUs at Siriraj Hospital between January 2020 and December 2021.

**Results:**

Of the 2,430 patients included in this study, 229 (9.4%) died; the mean age was 54 years, 40% were men, 81% had at least one comorbidity, and 13% required intensive care unit (ICU). Favipiravir (86%) was the main antiviral treatment. Corticosteroids and rescue anti-inflammatory therapy were used in 74 and 6%, respectively. Admission to the ICU was the only factor associated with reduced mortality [odds ratio (OR) 0.01, 95% confidence interval (CI) 0.01–0.05, *P* < 0.001], whereas older age (OR 14.3, 95%CI 5.76–35.54, *P* < 0.001), high flow nasal cannula (HFNC; OR 9.2, 95% CI 3.9–21.6, *P* < 0.001), mechanical ventilation (OR 269.39, 95%CI 3.6–2173.63, *P* < 0.001), septic shock (OR 7.79, 95%CI, 2.01–30.18, *P* = 0.003), and hydrocortisone treatment (OR 27.01, 95%CI 5.29–138.31, *P* < 0.001) were factors associated with in-hospital mortality.

**Conclusion:**

The overall mortality of hospitalized patients with COVID-19 was 9%. The only factor associated with reduced mortality was admission to the ICU. Therefore, appropriate selection of patients for admission to the ICU, strategies to limit disease progression and prevent intubation, and early detection and prompt treatment of nosocomial infection can improve survival in these patients.

## Introduction

COVID-19 (Corona virus disease 2019) is an emerging disease declared by the WHO (World Health Organization) as a Public Health Emergency of International Concern in January 2020. Since the first case reported from China in December 2019, more than 500 million people have been infected worldwide, with an overall mortality rate of 1.14% (1), a mortality rate 17–28% for hospitalized patients, and 49–60% for mechanically ventilated patients (2–10). Risk factors for increased mortality from previous studies included older age, pre-existing medical illnesses, high Sequential Organ Failure Assessment (SOFA) score, receipt of IMV, acute respiratory distress syndrome (ARDS), and elevated D-dimer (2, 6, 9, 11, 12). However, most data were reported from China and developed countries, while the epidemiological data for COVID-19 diseases from low- and middle-income countries are lacking. The limitation of healthcare resources and treatment capacity may impact patient outcomes. This study aimed to determine the characteristics and outcomes and identify risk factors for in-hospital mortality for hospitalized COVID-19 patients in Thailand.

## Materials and methods

### Study design and population

This is a retrospective cohort study conducted at Siriraj Hospital, a tertiary care academic hospital in Bangkok, Thailand. The study protocol was approved by the Human Research Protection Unit of Siriraj Hospital Faculty of Medicine, Mahidol University. The requirement for written informed consent was waived.

In Thailand, the COVID-19 pandemic has occurred since January 2020. All COVID-19 patients who require admission to our hospital are treated in cohort wards and intensive care units (ICU) with modified airborne infection isolation rooms (AIIR). The patients requiring vasopressors, high flow nasal cannula (HFNC), non-invasive ventilation (NIV), or invasive mechanical ventilation (IMV) were admitted to the ICUs when beds were available. Guideline for critical care management of severe COVID-19 patients in our hospital has been previously published (13–15). Moreover, the recommendation for antiviral and anti-inflammatory therapies for COVID-19 patients in Thailand have been changed regularly according to the updated published data (16, 17). Since the publication of the preliminary report of the Randomized Evaluation of COVID-19 Therapy (RECOVERY) trial (18), dexamethasone has been recommended for all patients with a resting oxygen saturation of < 96% or a reduction in oxygen saturation of ≥ 3% after exercise-induced desaturation or those requiring respiratory support. However, before the publication of the RECOVERY study, some critically ill patients who had rapidly worsening respiratory symptoms with evidence of hyperinflammation and were less likely to have superimposed bacterial infections received corticosteroid therapy as previously described (13).

In this study, we investigated all adult patients aged ≥ 18 years diagnosed with COVID-19 and admitted to the cohort wards and ICUs at Siriraj Hospital from January 2020 to December 2021. The diagnosis of COVID-19 disease was confirmed by detecting severe acute respiratory syndrome coronavirus-2 (SARS-CoV-2) from any respiratory specimens by reverse transcription-polymerase chain reaction (RT-PCR). Patients who were readmitted due to diagnoses other than reinfection of COVID-19 were excluded.

### Data collection

We initially obtained data from electronic medical records (EMRs) in collaboration with Siriraj Informatics and Data Innovation Center (SiData +) and then manually reviewed the EMRs of all patients to verify the diagnosis of COVID-19 infections and recorded the data that could not be collected electronically.

The data collected included demographic data of the patient, comorbidities, vital signs, and laboratory results at admission, antiviral and anti-inflammatory medications used for the treatment of COVID-19 disease, admission to the ICU, the requirement for renal replacement therapy (RRT) and respiratory support, including oxygen therapy, HFNC, NIV, IMV. The respiratory rate oxygenation index (ROX), the ratio of oxygen saturation measured by pulse oximetry (SpO_2_)/FiO_2_ to respiratory rate, was also calculated in all patients (19).

Patient outcomes included survival at hospital discharge, ICU and hospital length of stay, complications during admission, including ARDS according to the Berlin Definition (20), nosocomial infections, septic shock, venous thromboembolism, and bleeding complications.

### Statistical analysis

Categorical variables were presented as numbers and column percentages, while continuous variables were presented as mean with standard deviation (SD), or median with 25th and 75th quartiles, as appropriate. Categorical variables were compared using Chi-square or Fisher exact tests, while continuous variables were compared using the *t*-test or Mann-Whitney *U*-test, as appropriate. A multivariate logistic regression model with a stepwise forward method was used to identify independent clinical risk factors associated with in-hospital mortality. We report risk factors with odds ratios (OR) and 95% confidence intervals (CI). All *p*-values < 0.05 were considered statistical significance. All statistical analyses were performed using IBM SPSS Statistics version 18 (SPSS, Inc., Chicago, IL, USA) (21).

## Results

### Study patients

**FIGURE 1:**
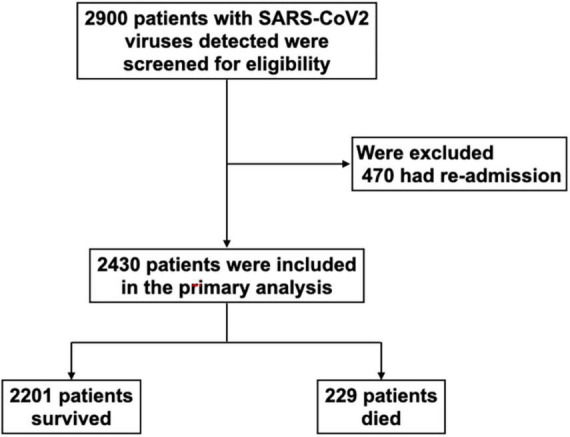
Study population. SARS-CoV-2, severe acute respiratory syndrome coronavirus-2.

**FIGURE 2:**
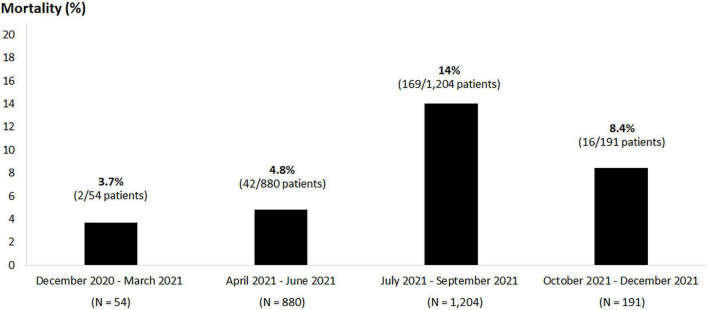
Trends in mortality from December 2020 to December 2021. Most cases (*n* = 2,329) were admitted from December 2020 to December 2021. The mortality rates changed over time; the highest mortality (14%) occurred at the same time as the highest admission rate (1,204 hospitalized patients) in July–September 2021. Only 101 patients were admitted between January and April 2020; no mortality was observed in that period, and no COVID-19 patient was hospitalized from May to November 2020.

From January 2020 to December 2021, 2,900 patients with SARS-CoV2 viruses detected by RT-PCR were admitted to the cohort wards and ICUs of Siriraj hospital. Of these patients, 470 were excluded due to readmission for diagnoses other than COVID-19 reinfections; the remaining 2,430 patients were included in the final analysis (Figure 1); 2,201 (91%) survived and 229 (9%) died. In Thailand, the first case of the delta variant was reported in December 2020, and the first case of the omicron variant was found in December 2021. In our cohort, most patients (*n* = 2,329) were admitted from December 2020 to December 2021. The mortality rates changed over time; the highest mortality (14%) occurred at the same time as the highest admission rate (1,204 hospitalized patients) in July–September 2021 (Figure 2). Only 101 patients were admitted between January and April 2020; no mortality was observed in that period, and no COVID-19 patient was hospitalized from May to November 2020.

### Baseline characteristics and laboratory results

The mean age of the hospitalized patients was 54 years, 40% were men and 19% had a body mass index (BMI) > 30 kg/m^2^. Most of the patients (81%) had at least one comorbid disease; 44% had hypertension, 26% had diabetes mellitus, 9% had chronic kidney disease, and 2% had chronic heart disease. The mean duration from the onset of symptoms to hospital admission was 5 days, and only 2% of the patients had hypotension [mean arterial pressure (MAP) < 65 mmHg] at admission. For patients receiving HFNC, the mean ROX index was 17.8. In the univariate analysis, non-survived patients were older [74 (13) vs. 52 (19) years, *P* < 0.001], and were more often men (58% vs. 37%, *P* < 0.001) and had hypertension (74% vs. 41%, *P* < 0.001), diabetes mellitus (32% vs. 25%, *P* = 0.045), chronic kidney disease (28% vs. 7%, *P* < 0.001), and chronic heart disease (5% vs. 1%, *P* < 0.001), required a higher FiO2 [0.5 (0.3) vs. 0.3 (0.2), *P* < 0.001], and had a lower ROX index [10.4 (6.7) vs. 18.5 (6.4), *P* < 0.001] compared to patients who were discharged alive. The BMI and duration from the onset of the symptoms to admission were not significantly different between the survived and non-survived patients (Table 1).

**TABLE 1:** Baseline characteristic within 24 h of hospital admission according to hospital mortality.

	Overall population	Survived	Deceased	*P*-value
**Demographics**	2,430	2,201	229	
Age, year	54.2 ± 19.8	52.1 ± 19.2	74.3 ± 13.1	<0.001
Male gender (%)	982 (40.4)	850 (38.6)	132 (57.6)	<0.001
Weight, kg	66.4 ± 17.4	66.1 ± 17.5	63.3 ± 15.1	0.006
Height, cm	160.7 ± 9.0	160.8 ± 9.1	160.5 ± 8.5	0.697
BMI, kg/m^2^	25.6 ± 6.0	25.7 ± 6.0	24.6 ± 5.9	0.007
BMI > 30 kg/m^2^	471 (19)	431 (20)	40 (18.1)	0.502
**Comorbidities**				
Diabetes (%)	640 (26.3)	567 (25.3)	73 (31.9)	0.045
Hypertension (%)	1,070 (44.2)	904 (41.1)	170 (74.2)	<0.001
Heart disease (%)	37 (1.5)	26 (1.2)	11 (4.8)	<0.001
CKD overall (%)	223 (9.2)	160 (7.3)	63 (27.5)	<0.001
Stage 3 (%)		79 (3.6)	35 (15.3)	<0.001
Stage 4 (%)		16 (0.7)	12 (5.2)	<0.001
Stage 5 (%)		65 (3)	16 (7)	0.001
Day onset of symptom, days	4.9 ± 3.8	4.9 ± 3.7	4.8 ± 4.2	0.126
Respiratory rate, per minute	22.2 ± 4.1	21.9 ± 3.4	25.2 ± 5.1	<0.001
Oxygen saturation, %	97.2 ± 2.8	97.3 ± 2.6	95.7 ± 4.2	<0.001
FiO2, %	0.32 ± 0.19	0.29 ± 0.16	0.53 ± 0.28	<0.001
ROX index	17.8 ± 6.9	18.51 ± 6.43	10.43 ± 6.67	<0.001
MAP, mmHg	96.1 ± 11.8	96.6 ± 11.5	91.1 ± 13.6	<0.001
MAP < 65 mmHg (*n* = 2,419)		0 (0%)	5 (2.2%)	<0.001

BMI, body mass index; CKD, chronic kidney disease; FiO2, fraction inspired oxygen; MAP, mean arterial pressure; ROX index, the ratio of pulse oximetry/fraction of inspired oxygen to respiratory rate.

For baseline laboratory results, patients who did not survive had slightly lower hemoglobin [12 (2) vs. 13 (2) g/dl, *P* < 0.001], platelet counts [206 (97) vs. 243 (92) × 10^3^/mm^3^, *P* < 0.001], and serum sodium [136 (6) vs. 137 (2) mEq/L, *P* < 0.001], bicarbonate [21 (4) vs. 22 (3) mEq/L, *P* < 0.001] and albumin level [3.1 (0.6) vs. 3.7 (0.6) g/dl, *P* < 0.001], but higher white blood cells [8.2 (4.8) vs. 6.8 (3.4) × 10^3^ cells/mm^3^, *P* < 0.001], serum creatinine [Cr; 1.9 (2.3) vs. 1.2 (1.8) mg/dl, *P* < 0.001], potassium [4.2 (0.7) vs. 3.9 (0.6) mEq/L, *P* < 0.001], total bilirubin [0.8 (1.3) vs. 0.5 (0.5) mg/dl, *P* = 0.024], aspartate aminotransferase [AST; 75 (130) vs. 41 (49) U/L, *P* = 0.002], alanine aminotransferase [ALT; 52 (95) vs. 37 (55) U/L, *P* = 0.003], C-reactive protein [CRP; 75 (64) vs. 35 (46) mg/L, *P* < 0.001], and procalcitonin level [3.3 (9.8) vs. 1.2 (3.6) mg/mL, *P* < 0.001]. There were no significant differences in hemoglobin A1c, serum chloride, and alkaline phosphatase levels (Table 2).

**TABLE 2:** Baseline laboratory data within 24 h of admission according to hospital mortality.

Variables	Overall (2,430)	Survived (2,201)	Deceased (229)	*P*-value
Hemoglobin, g/dl	12.7 ± 2.0	12.74 ± 1.97	11.90 ± 2.45	<0.001
Hematocrit, %	38.5 ± 5.7	38.7 ± 5.5	36.4 ± 7.0	<0.001
Hematocrit < 36% (*n* = 2,259)	662 (29.3%)	571 (27.6%)	91 (44.6%)	<0.001
WBC,/mm^3^	6,880 ± 3,567	6,770 ± 3,431	8,240 ± 4,833	<0.001
WBC > 12,000 (*n* = 1,931)	155 (8.0%)	124 (69%)	31 (22.6%)	<0.001
Platelet, mm^3^	240,060 ± 93,241	243,450 ± 92,250	205,910 ± 97,017	<0.001
Platelet < 150,000 (*n* = 2,259)	307 (13.6%)	240 (11.7%)	67 (32.8%)	<0.001
HbA1c	7.0 ± 1.9	7.02 ± 1.92	7.04 ± 1.85	0.926
BUN, mg/dL	18.8 ± 18.1	17.0 ± 15.4	36.3 ± 29.4	<0.001
Cr, mg/Dl	1.24 ± 1.83	1.17 ± 1.77	1.91 ± 2.26	<0.001
Cr > 2 mg/dL (*n* = 2,174)	150 (6.9%)	110 (5.6%)	40 (20.2%)	<0.001
Sodium, mEq/L (*n* = 2,218)	137.1 ± 4.3	137.1 ± 1.77	136.1 ± 6.3	0.001
Sodium > 145 mEq/L	28 (1.3%)	14 (0.7%)	14 (6.5%)	<0.001
Sodium < 135 mEq/L	476 (21.3%)	397 (19.8%)	79 (35.3%)	<0.001
Potassium, mEq/L (*n* = 2,217)	3.9 ± 0.6	3.9 ± 0.6	4.2 ± 0.7	<0.001
Potassium > 5 mmol/L	73 (3.3%)	56 (2.8%)	17 (7.9%)	<0.001
Potassium < 3 mmol/L	33 (1.5%)	30 (1.5%)	3 (1.4%)	0.906
Chloride, mEq/L	100.6 ± 4.9	100.6 ± 4.7	100.2 ± 6.7	0.227
HCO_3_, mEq/L	22.4 ± 3.4	22.6 ± 3.3	20.6 ± 4.2	<0.001
HCO_3_ < 20 mEq/L (*n* = 2,214)	380 (17.2%)	299 (15%)	81 (37.7%)	<0.001
CRP	38.4 ± 49.2	35.1 ± 46.3	74.9 ± 63.6	<0.001
Procalcitonin	1.46 ± 5.02	1.16 ± 3.59	3.26 ± 9.84	<0.001
AST, IU/mL	43.5 ± 59.2	40.9 ± 48.5	75.2 ± 130.0	0.002
ALT, IU/mL	37.7 ± 58.9	36.6 ± 54.9	51.5 ± 94.5	0.003
ALP, IU/mL	90.9 ± 61.9	89.8 ± 61.8	99.3 ± 62.9	0.58
Albumin, g/dL	3.6 ± 0.6	3.72 ± 0.59	3.12 ± 0.61	<0.001
Albumin < 3 g/dL (*n* = 917)	122 (13.3%)	81 (10.4%)	41 (36%)	<0.001
Total bilirubin, mg/dL	0.57 ± 0.62	0.53 ± 0.45	0.84 ± 1.33	0.024
Total bilirubin > 3 mg/dL (*n* = 804)	8 (0.9%)	5 (0.7%)	3 (3.1%)	0.06
Direct bilirubin, mg/dL	0.31 ± 0.54	0.27 ± 0.34	0.61 ± 1.22	0.008

AST, aspartate aminotransferase; ALT, alanine aminotransferase; ALP, alkaline phosphatase; BUN, blood urea nitrogen; Cr, creatinine; CRP, C-reactive protein; WBC, white blood cell.

### Treatments and outcomes

Eighty-one percent of patients received respiratory support; 49% required low flow oxygen via nasal cannula or non-rebreathing mask with a reservoir bag, 25% required HFNC or NIV, and 7% required IMV. However, admission to the ICU was available for only 307 patients (13%). RRT was used in 90 patients (4%). Favipiravir (86%) was the most common antiviral medication, followed by remdesivir (9%) and lopinavir boosted with ritonavir (2%). Corticosteroids were administered to 1,799 patients (74%); dexamethasone (67%) was the most common type of corticosteroids used in patients with COVID-19, followed by methylprednisolone (3%) and hydrocortisone (3%). Rescue anti-inflammatory therapies with tocilizumab (4%) or baricitinib (2%) were required in 149 patients (6%). Of these patients, 113 survived (76%), and 36 died (24%; Table 3).

**TABLE 3:** Treatment and clinical outcomes of according to hospital mortality.

Variables	Overall (2,430)	Survived (2,201)	Deceased (229)	*P*-value
ICU admission, %	307 (13%)	233 (10.6%)	74 (31.6%)	<0.001
ICU LOS, days	9.0 + 7.4	7.5 + 6.6	14.1 + 7.6	<0.001
Hospital LOS, days	8.6 + 6.2	8.1 + 5.8	12.8 + 8.0	<0.001
**Respiratory support**				
Cannula, %	1,060 (44%)	912 (41.4%)	148 (64.6%)	<0.001
Mask with bag, %	133 (5%)	90 (4.1%)	43 (18.8%)	<0.001
HFNC, %	605 (25%)	418 (19%)	187 (81.7%)	<0.001
Mechanical ventilation, %	178 (7%)	79 (36%)	99 (43.2%)	<0.001
Renal replacement therapy	90 (4%)	63 (2.8%)	27 (11.8%)	<0.0001
**Antiviral therapy**				
Favipiravir, %	2,095 (86.2%)	1,883 (84.2%)	212 (92.6)	0.001
Remdesivir, %	230 (9.5%)	169 (7.7%)	61 (26.6%)	<0.001
Lopinavir/ritonavir, %	60 (2.5%)	58 (2.6%)	2 (2.9%)	0.102
**Anti-inflammatory therapy**				
Tocilizumab, %	103 (4%)	75 (3.4%)	28 (12.2%)	<0.001
Baricitinib, %	46 (2%)	38 (1.7%)	8 (3.5%)	0.06
Dexamethasone, %	1,639 (67%)	1,421 (64.8%)	218 (95.2%)	<0.001
Methylprednisolone, %	78 (3%)	54 (2.5%)	24 (10.5%)	<0.001
Hydrocortisone, %	82 (3%)	12 (0.5%)	70 (30.6%)	<0.001
**Complications**				
Acute respiratory distress syndrome, %	114 (4.7%)	51 (2.34%)	63 (22.5%)	<0.001
Septic shock, %	100 (4.1%)	21 (1%)	79 (34.5%)	<0.001
Nosocomial infection, %	155 (6.4%)	78 (3.5%)	77 (33.6%)	<0.001
Pulmonary embolism, %	34 (1.4%)	21 (1%)	13 (5.7%)	<0.001
Bleeding, %	15 (0.6%)	9 (0.4%)	6 (2.6%)	<0.001

AST, aspartate aminotransferase; ALT, alanine aminotransferase; ALP, alkaline phosphatase; BUN, blood urea nitrogen; Cr, creatinine; CRP, C-reactive protein; WBC, white blood cell.

Overall in-hospital mortality was 9%; the mortality rate increased to 31% (187 of 605) in patients requiring HFNC or NIV, and 56% (99 of 178) in patients requiring IMV. The mortality rate for patients admitted to the ICU was 24% (74 of 307). Deceased patients were admitted more frequently to the ICU and received antiviral and anti-inflammatory medications, respiratory support, and RRT than survived patients (Table 3). Nosocomial infections (6.4%) were the most common hospital complications, followed by ARDS (4.7%), septic shock (4.1%), pulmonary embolism (1.4%), and bleeding complications (0.6%). All complications occurred more frequently in patients who did not survive than in those who survived.

### Factors associated with hospital mortality

In a multivariate logistic regression analysis, the only factor related to mortality reduction in mortality was admission to the ICU (OR 0.01, 95%CI 0.01–0.05, *P* < 0.001). Factors associated with increased mortality included age > 65 years (OR 14.3, 95%CI 5.8–35.5, *P* < 0.001), thrombocytopenia (platelet < 150 × 10^3^/mm^3^; OR 2.4, 95%CI 1.1–5.5, *P* = 0.034), renal dysfunction (Cr > 2 mg/dL; OR 6.2, 95%CI 2.3–16.6, *P* < 0.001), ROX index < 12 (OR 2.32, 95%CI 1.0–5.0, *P* = 0.033), MAP < 90 mmHg (OR 4.8, 95%CI 2.3–4.9, *P* < 0.001), the use of respiratory support with low flow O_2_ through a non-rebreathing mask with a reservoir bag (OR 4.5, 95%CI 1.7–12.0, *P* < 0.001), non-invasive respiratory support (HFNC or NIV, OR 9.2, 95%CI 3.9–21.6, *P* < 0.001), and IMV (OR 269.4, 95%CI 33.6–2173.6, *P* < 0.001), receiving hydrocortisone (OR 27.0, 95%CI 5.3–138.3, *P* < 0.001) and occurrence of septic shock (OR 7.8, 95%CI 2.0–30.2, *P* < 0.001; Table 4).

**TABLE 4:** Univariate and multivariate analysis to predict hospital mortality.

Variables	Univariate		Multivariate	
	Odd (95%CI)	*P*-value	Odd (95%CI)	*P*-value
**Baseline parameters**				
Age > 65 years	8.41 (6.12–11.58)	<0.001	14.30 (5.76–35.54)	<0.001
Male gender	2.16 (1.64–2.85)	<0.001	−	−
Diabetes mellitus	1.35 (1.01–1.81)	0.045	−	−
Hypertension	4.13 (3.04–5.63)	<0.001	−	−
Heart disease	4.22 (2.06–8.66)	<0.001	−	−
Chronic kidney disease stage 3–5	4.84 (3.48–6.75)	<0.001	−	−
Hematocrit < 36%	2.09 (1.56–2.80)	<0.001	−	−
Platelet < 150,000/mm^3^	3.70 (3.68–5.10)	<0.001	2.43 (1.07–5.53)	0.034
WBC > 12,000/mm^3^	3.94 (2.54–6.11)	<0.001	−	−
Creatinine > 2 mg/dL	4.30 (2.89–6.39)	<0.001	6.22 (2.33–16.60)	<0.001
Sodium > 145 mEq/L	9.90 (4.65–21.05)	<0.001	−	−
Sodium < 135 mEq/L	2.21 (1.64–2.99)	<0.001	−	−
Potassium > 5 mEq/L	2.98 (1.70–5.23)	<0.001	−	−
Bicarbonate < 20 mEq/L	3.44 (2.54–4.65)	<0.001	−	−
Albumin < 3 g/dL	5.01 (3.20–7.82)	<0.001	−	−
AST > 40 IU/mL	3.56 (2.51–5.04)	<0.001	−	−
Total bilirubin > 3 mg/dL	4.74 (1.12–20.16)	0.020	−	−
Respiratory rate > 20/min	5.12 (3.72–7.06)	<0.001	−	−
Pulse oximetry < 95%	3.66 (2.67–5.01)	<0.001	−	−
ROX index < 12	8.69 (6.45–11.69)	<0.001	2.32 (1.01–5.01)	0.033
MAP < 90 mmHg	2.26 (1.72–2.98)	<0.001	4.75 (2.28–4.88)	<0.001
**Treatment parameters**				
ICU admission	3.95 (2.90–5.39)	<0.001	0.01 (0.01–0.05)	<0.001
Mask with bag	5.42 (3.66–8.03)	<0.001	4.51 (1.70–11.97)	<0.001
High flow nasal cannula	18.97 (13.35–26.70)	<0.001	9.23 (3.94–21.60)	<0.001
Mechanical ventilation	20.46 (14.49–28.87)	<0.001	269.39 (33.6–2173.63)	<0.001
Renal replacement therapy	4.54 (2.83–1.28)	<0.001	−	−
Favipiravir	2.34 (1.41–3.89)	<0.001	−	−
Remdesivir	4.37 (3.13–6.09)	<0.001	−	−
Tocilizumab	3.95 (2.50–6.24)	<0.001	−	−
Baricitinib	2.06 (0.95–4.76)	0.06	−	−
Dexamethasone	10.45 (5.83–19.82)	<0.001	−	−
Methylprednisolone	4.66 (2.82–7.69)	<0.001	−	−
Hydrocortisone	80.31 (42.64–151.27)	<0.01	27.04 (5.29–138.31)	<0.001
**Complications**				
ARDS	16.00 (10.71–23.91)	<0.001	−	−
Septic shock	54.67 (32.87–90.94)	<0.001	7.79 (2.01–30.18)	0.003
Nosocomial infection	13.79 (9.67–16.67)	<0.001	−	−
Pulmonary embolism	6.25 (3.09–12.65)	<0.001	−	−
Bleeding	6.55 (2.31–18.58)	<0.001	−	−

A predictive model including only baseline parameters was shown in Supplementary Table 1. Similar baseline parameters including age > 65 years, platelet < 150 × 10^3^/mm^3^, Cr > 2 mg/dL, ROX index < 12, and MAP < 90 mmHg were persistently associated with increased mortality. Moreover, abnormal liver test namely serum albumin < 3 g/dL, AST > 40 IU/mL, and total bilirubin > 3 mg/dL were independently associated with greater mortality.

## Discussion

In this large cohort of patients hospitalized for COVID-19 infections in Thailand, the overall mortality rate was 9%; the rate was higher in patients requiring admission to the ICU (24%) and IMV (56%). In multivariate analysis, admission to the ICU was the only factor associated with a reduction in mortality. Patients who were elderly, required high-level respiratory support, especially those with a lower ROX index, had renal dysfunction and thrombocytopenia at admission, were complicated by septic shock, and received hydrocortisone were at high risk of mortality in hospital.

The overall mortality of 9% in our cohort was lower than previously reported during the early stage of the pandemic (2, 3), but the mortality in patients who required admission to the ICU (24%) and IMV (56%) was comparable (3–5, 7–9). In previous studies, the mortality rate of hospitalized patients with COVID-19 ranged from 17 to 28% (2–4, 6, 18), and the mortality for ICU patients was 24–62% and for mechanically ventilated patients was 49–62% (3–5, 7–11).

A better understanding of the natural history of the disease and the availability of antiviral and anti-inflammatory therapies can lead to a reduction in mortality over time. Prior studies were carried out during the early stage of the pandemic, January–April 2020, in which information on the natural course and effective treatment strategies for emerging diseases was limited (3, 4, 6), while our study period was longer (January 2020–December 2021) and included the later stage of the pandemic when more scientific knowledge about COVID-19 was available (18, 22–27). The difference in mortality of hypoxemic COVID-19 who did not require mechanical ventilation at admission was also found in two randomized controlled trials conducted in different periods. The RECOVERY trial revealed the effectiveness of dexamethasone in COVID-19 and was carried out between March and June 2020. That trial reported that the mortality at 28 days was 23% in the dexamethasone group and 26% in the usual care group (18). The Adaptive COVID-19 Treatment Trial 4 (ACTT-4) compared baricitinib and dexamethasone in combination with remdesivir for the treatment of hospitalized patients with COVID-19 was carried out from December 2020 to April 2021. ACTT-4 reported that mortality at 60 days was only 7 and 8% in the baricitinib and dexamethasone groups, respectively (28).

The different variants of the virus and the availability of COVID-19 vaccines may be the other important reasons for the decreased mortality in our cohort (29). A prior study also reported a lower fatality of COVID-19 patients during the outbreak of the omicron variant compared to that of the delta and beta variants (30). At the end of 2021, 63% of the Thai population had already received at least two doses of vaccines (31), and all types of COVID-19 vaccines are proven to reduce the risk of developing severe disease and death (32–36). Unfortunately, information on COVID-19 vaccination status for our individual patients was not available, so the influence of the vaccination status on hospital mortality could not be determined.

This study also revealed the survival benefit of admission to the ICU for patients with severe COVID-19 (Table 4). Of the 307 patients admitted to the ICU, 76% were discharged alive. According to our results, patients who needed high-level respiratory support, HFNC or IMV, and who had septic shock and renal failure were at the highest risk of death; these patients should be the main priority for admission to the ICU. However, we also demonstrated the shortage of cohort ICU beds in our hospital, since almost one-third of our patients received HFNC or IMV, but only 13% of all patients were treated in the ICU. Therefore, increasing the capacity of the ICU with AIIR is an essential plan to prepare for the next pandemic.

Previous studies have reported an association between the degree of hypoxemia and increased mortality; our results supported this finding (4, 9, 11, 37). The lower ROX index and use of respiratory supports represented more severe hypoxemia in our patients. Since the risk of death was much higher in patients requiring IMV than in those requiring HFNC and low-flow O_2_ therapy (Table 4), strategies to limit disease progression and prevent intubation, including early antiviral treatment for patients at high risk of disease progression, and rescue anti-inflammatory therapy with tocilizumab or baricitinib for those who had rapid worsening despite corticosteroid treatment, appear reasonable (25–27, 38).

Hydrocortisone is the only type of corticosteroid found to be associated with mortality in this study (Table 4). In contrast to dexamethasone, no prior research has demonstrated the benefit of hydrocortisone in patients with COVID-19 (39, 40). We found that septic shock was associated with high mortality in the multivariate model. This association between hydrocortisone and mortality could be explained by the fact that in our hospital hydrocortisone was used primarily in patients with septic shock who received high-dose vasopressor treatment. An observational study in Argentina also found that septic shock and refractory hypoxemia were the major causes of death in patients with COVID-19 requiring IMV, and the use of vasopressors on admission was an independent predictor of hospital mortality (10), the rate of nosocomial infection in our cohort was 6% (155 of 2,430) and 65% (100 of 155) of these patients developed septic shock (Table 3). Therefore, early detection and treatment of superimposed bacterial infections are essential to improve patient outcomes.

The associations between increased mortality and older age, thrombocytopenia, and renal dysfunction on admission in patients with COVID-19 have been reported in previous studies (2, 4, 8, 11, 41–43), but older age is not modifiable, and thrombocytopenia and renal dysfunction appear to be attributable to the severity of the disease (44–47). Thrombocytopenia, defined as platelet counts < 150 × 10^3^/mm^3^, is common in COVID-19 disease. The proposed pathophysiological mechanisms include reducing platelet synthesis from the bone marrow due to direct viral infection or cytokine storm, increasing platelet destruction by the immune system, and increased platelet consumption resulting from aggregation and formation of microthrombi in damaged lung parenchyma and pulmonary endothelial cells (48). The possible pathophysiology of acute kidney injury (AKI) in COVID-19 consists of direct effects of the virus that causes endothelial and tubular epithelial damage and indirect impact from volume depletion, nephrotoxic drugs, sepsis-associated AKI from superimposed bacterial infection, increased renal venous pressure complicated from mechanical ventilation with high intrathoracic pressure, organ crosstalk from lung injury or cardiorenal syndrome, and rhabdomyolysis (46, 47). Patients with any of these factors need close observation for severe disease.

The strengths of our study are that we reported the characteristics and outcomes of a relatively large cohort of hospitalized patients with COVID-19 and performed a multivariate analysis to determine the independent factors associated with in-hospital mortality. We also performed manual searches on the EMR to verify the diagnosis of COVID-19 infection in all patients and to obtain the data that could not be collected electronically. We also acknowledge several limitations of this study. First, this is a single-center study, which may limit the generalizability of our findings. Second, we did not have data on underlying chronic lung disease, SOFA score, and D-dimer level, which increased mortality risks in previous studies. Third, concurrent life-threatening medical or surgical diseases necessitating urgent treatment were not recorded; these conditions may impact patient outcomes. Fourth, we were unable to obtain clinical outcomes after hospital discharge. Finally, similar to other observational and retrospective studies, unmeasured confounders may affect the results.

## Conclusion

The overall mortality of hospitalized COVID-19 patients in Thailand was 9%. Admission to the ICU was the only protective factor. Patients who received invasive or non-invasive respiratory support and were complicated by septic shock had the highest risk of death. Therefore, appropriate patient selection for admission to the ICU, strategies to limit disease progression and prevent intubation, and early detection and prompt treatment of nosocomial infection may improve survival. Although unmodifiable, elderly patients and those with thrombocytopenia and renal dysfunction need close observation for the development of severe disease.

## Data availability statement

The raw data supporting the conclusions of this article will be made available by the authors, without undue reservation.

## Ethics statement

The studies involving human participants were reviewed and approved by the Human Research Protection Unit of Siriraj Hospital Faculty of Medicine, Mahidol University (Certificate of Approval no. Si 335/2020). Written informed consent for participation was not required for this study in accordance with the national legislation and the institutional requirements.

## Author contributions

TN had full access to all data in the study and takes responsibility for the integrity of the data and the accuracy of the data analysis, designed the study, performed manual searches for additional data collection and interpretation, drafted and revised the manuscript. TV designed the study, reviewed, interpreted the data, drafted, and revised the manuscript. ST designed the study, performed the statistical analysis, drafted, and revised the manuscript. AD, TP, RW, RR, PP, PT, AP, and CP assisted in data collection, data interpretation, and critically reviewed the manuscript. All authors have read and approved the final manuscript and agreed to be responsible for all aspects of the work.
