# Supplementary material for: Characteristics, outcomes, and risk factors for in-hospital mortality of COVID-19 patients: A retrospective study in Thailand
Source: Front Med (Lausanne). 2023 Jan 4;9:1061955. doi: 10.3389/fmed.2022.1061955 (PMC9846200; doi:10.3389/fmed.2022.1061955)
Supplement: Supplementary file 1 [file Table_1.DOCX]

**Supplemental Materials**

**Characteristics, Outcomes, and Risk Factors for In-Hospital Mortality of COVID-19 Patients: A Retrospective Study in Thailand**

Thummaporn Naorungroj, MD; Tanuwong Viarasilpa*, MD; Surat Tongyoo, MD; Aeckapholpholladet Detkaew, RN, Thanchanok Pinpak, MD; Rawish Wimolwattanaphan, MD; Ranistha Ratanarat, MD; Panuwat Promsin, MD; Preecha Thamrongpiroj, MD; Akekarin Phumpichet, MD; Chairat Permpikul, MD

**Author Affiliations**

Division of Critical Care, Department of Medicine Siriraj Hospital, Mahidol University, Bangkok, Thailand 10700

**REVISION 1**

**Corresponding Author**

Tanuwong Viarasilpa

Division of Critical Care, Department of Medicine, Siriraj Hospital

2 Wanglang Road, Bangkok, Thailand 10700

Tel. 02-419-8597

E-mail: tanuwong.via@mahidol.ac.th

| **Supplemental Table 1: Multivariate Analysis Included Only Baseline Parameters** | | |
| --- | --- | --- |
| **Variables** | **Multivariate** | |
|  | **Odd (95%CI)** | **P-value** |
| **Baseline Parameters** |  |  |
| Age > 65 years | 13.39 (6.34 – 28.26) | <0.001 |
| Male gender | - | - |
| Diabetes Mellitus | - | - |
| Hypertension | - | - |
| Heart Disease | - | - |
| Chronic Kidney Disease stage 3-5 | - | - |
| Hematocrit < 36% | - | - |
| Platelet < 150,000/mm3 | 3.56 (1.81 – 6.97) | <0.001 |
| WBC > 12,000/mm3 | - | - |
| Creatinine > 2 mg/dL | 3.81 (1.33 – 10.92) | 0.013 |
| Sodium > 145 mEq/L | - | - |
| Sodium < 135 mEq/L | - | - |
| Potassium > 5 mEq/L | - | - |
| Bicarbonate < 20 mEq/L | - | - |
| Albumin < 3 g/dL | 2.54 (1.27 – 5.08) | 0.009 |
| AST > 40 IU/mL | 2.06 (1.11 – 3.83) | 0.022 |
| Total bilirubin > 3 mg/dL | 8.04 (1.09 – 59.28) | 0.041 |
| Respiratory rate > 20/min | - | - |
| Pulse oximetry < 95% | - | - |
| ROX index < 12 | 7.06 (3.37 – 14.83) | <0.001 |
| MAP < 90 mmHg | 2.98 (1.64 – 5.41) | <0.001 |
